# Computing the Viscosity of Supercooled Liquids: Markov Network Model

**DOI:** 10.1371/journal.pone.0017909

**Published:** 2011-03-25

**Authors:** Ju Li, Akihiro Kushima, Jacob Eapen, Xi Lin, Xiaofeng Qian, John C. Mauro, Phong Diep, Sidney Yip

**Affiliations:** 1 Department of Materials Science and Engineering, University of Pennsylvania, Philadelphia, Pennsylvania, United States of America; 2 Department of Nuclear Science and Engineering and Department of Materials Science and Engineering, Massachusetts Institute of Technology, Cambridge, Massachusetts, United States of America; 3 Department of Nuclear Engineering, North Carolina State University, Raleigh, North Carolina, United States of America; 4 Division of Materials Science and Engineering, Department of Mechanical Engineering, Boston University, Boston, Massachusetts, United States of America; 5 Science & Technology Division, Corning Incorporated, Corning, New York, United States of America; Massachusetts Institute of Technology, United States of America

## Abstract

The microscopic origin of glass transition, when liquid viscosity changes continuously by more than ten orders of magnitude, is challenging to explain from first principles. Here we describe the detailed derivation and implementation of a Markovian Network model to calculate the shear viscosity of deeply supercooled liquids based on numerical sampling of an atomistic energy landscape, which sheds some light on this transition. Shear stress relaxation is calculated from a master-equation description in which the system follows a transition-state pathway trajectory of hopping among local energy minima separated by activation barriers, which is in turn sampled by a metadynamics-based algorithm. Quantitative connection is established between the temperature variation of the calculated viscosity and the underlying potential energy and inherent stress landscape, showing a different landscape topography or “terrain” is needed for low-temperature viscosity (of order 10^7^ Pa·s) from that associated with high-temperature viscosity (10^−5^ Pa·s). Within this range our results clearly indicate the crossover from an essentially Arrhenius scaling behavior at high temperatures to a low-temperature behavior that is clearly super-Arrhenius (fragile) for a Kob-Andersen model of binary liquid. Experimentally the manifestation of this crossover in atomic dynamics continues to raise questions concerning its fundamental origin. In this context this work explicitly demonstrates that a temperature-dependent “terrain” characterizing different parts of the same potential energy surface is sufficient to explain the signature behavior of vitrification, at the same time the notion of a temperature-dependent effective activation barrier is quantified.

## Introduction

A longstanding problem in the molecular theory of transport is the calculation of the temperature variation of the shear viscosity of highly viscous liquids [1]. It is well known that below a certain temperature range the shear stress relaxation becomes too slow for molecular dynamics (MD) simulation [2] to directly address experimental data [3], [4] that vary by 15 orders of magnitude. Recently we proposed a modified Green-Kubo method for the viscosity using a master-equation formulation with transition state pathway (TSP) sampling. Two versions have evolved from this approach, a heuristic model of an effective temperature-dependent activation barrier [5], [6] and a Network model in the framework of linear response theory. Both make use of TSP trajectories [5], [6] sampled by a metadynamics [7] activation-relaxation algorithm as the input.

In this paper we analytically derive and provide numerical details of the implementation of the Network model. The calculation shows that more than one typical energy landscape (“terrain”) is needed in to span the full temperature range of existing data. For liquids and modestly supercooled liquids, terrains of shallow minima and low activation energies lead to viscosity variation in agreement with molecular dynamics simulations. For highly supercooled liquids deep minima and large activation energies give results that compare well with experimental data [3], [4]. The demonstration of a method to calculate the viscosity over a range of 10 or more orders of magnitude means we now have an explanation of the molecular origin of the phenomenon of dynamic crossover in the temperature variation of the viscosity of glass-forming liquids. The crossover from Arrhenius behavior at high temperature to super-Arrhenius behavior across a characteristic temperature has been recently emphasized as a significant universal feature of glass transition [8] after an extensive analysis of the data on 84 liquids.

## Methods

### Derivation of Markov Network Model

**Figure 1:**
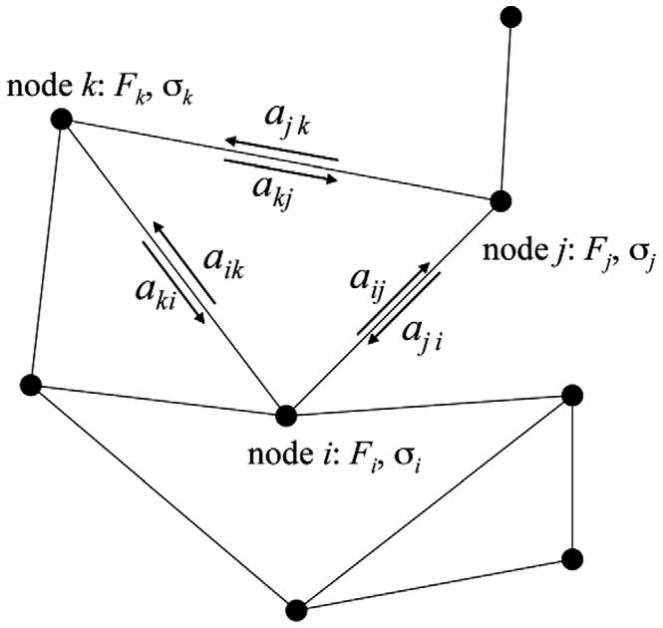
Network of coupled energy basins with free energy 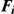, Eq. (1), average shear stress 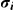, Eq. (2), and pairwise transition rates 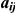, Eq. (3).

We recast the Green-Kubo theory of viscosity [9] into a form where the kinetics of stress relaxation is described by a Markov system of nodes, with pair-wise transition rates specified by an activation energy in standard transition state theory. Consider a system of *N* interacting particles **x** ^3*N*^ within volume 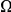 at temperature *T*. The system is characterized by an ensemble of basins (nodes in a Markov network, see Fig. 1) indexed by *i*, with associated constrained free energy,
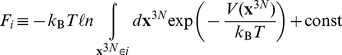
(1)where *V*(**x** ^3*N*^) is the interatomic potential and the integration is over basin *i* configurational states only. One can define an “inherent stress” for basin *i*,
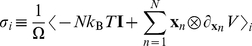
(2)where **I** is 3×3 identity matrix, and the thermal averaging of Virial stress <>*_i_* is performed within basin *i* configurational states only. From now on we will only consider the shear component (off-diagonal component) of stress, and regard the inherent stress 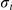 as a scalar quantity.

We then assume the basins are connected by a set of pairwise “bridges”, with transition rate
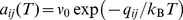
(3)connecting basin *i* and *j*, where *ν*_0_ is a trial frequency, and 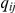 is the activation barrier separating basin *j* from basin *i*. The Network model is thus specified by the nodal energies, stresses, and the Markov transition rates 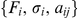. To use this model to calculate the shear viscosity 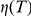 of a liquid, we recall the Green-Kubo formalism in linear response theory where 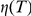 is given by the expression,
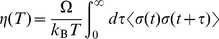
(4)where 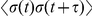 is the time-dependent shear stress correlation function. Since at any given time the system has to reside in one of the basins, we can write the shear stress coarse-grained in time as
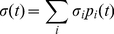
(5)with 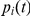 being a state-residence function, equal to unity if the system is in basin *i* at time *t*, and zero otherwise. We expect the coarse-graining scheme (5) to be asymptotically correct in the limit of long residence times, i.e. if the basin hoppings are “rare events”.

We then introduce a conditionally averaged stress, if the system is in basin *i* at time 0,
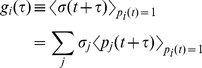
(6)with *t*-dependence dropped in the above, utilizing the Markovian (or “memoryless”) assumption about the basin hoppings. Again, we expect (6) to be asymptotically correct in the limit of “rare-event” hoppings. The stress correlation function then becomes an average over nodes
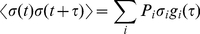
(7)with
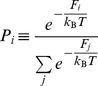
(8)being the probability that the system is in state *i* at any given time. Thus the viscosity also becomes a nodal average
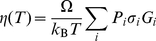
(9)with
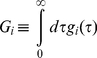
(10)


The function 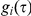 has the physical interpretation of the average shear stress at time 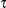 given the system was in state *i* at an earlier time 0. Based on the Markovian “memoryless” assumption, it should satisfy the balance equation,
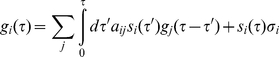
(11)


Here
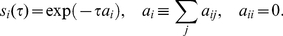
(12)is the probability that the system will stay at node *i* during a time interval 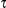. The terms in the *j*-sum account for the contribution from processes where the system has moved from state *i* to a number of intermediate states, while the last term in (11) is the contribution if the system remains in state *i* during the time interval 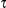.

Eq.(11) is a linear integral equation that can be readily solved. We can perform Laplace transformation 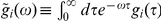 on both sides of (11). In frequency space it reads
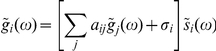
where 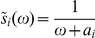, or
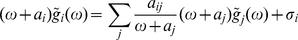
(13)The solution to Eq.(13) is just
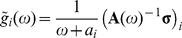
(14)in matrix-vector notation, where 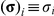 and
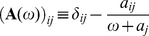
(15)the vector **σ** and matrix **A**(*ω*) being *M*×1 and *M*×*M*, respectively, if we consider a Markov network of *M* basins.

Since 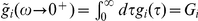, we obtain a closed-form “fluctuation-dissipation” expression for the shear viscosity,
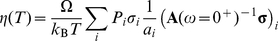
(16)where 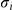 is understood to be a stress fluctuation, i.e. there needs to be
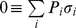
(17)sum rule. In an actual numerical calculation, if the sampled 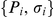 does not give zero mean, the non-zero mean needs to be subtracted off from 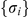 to make sure Eq.(17) constraint is satisfied.

Eq.(16) is a coarse-grained Green-Kubo expression where the viscosity 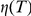 is explicitly resolved as a coarse-grained shear stress correlation, the product of two “inherent shear stresses” modulated by a propagator matrix **A**. Dimensionally speaking, Eq.(16) reminds us that the viscosity unit of stress-time [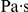] is a product of stress fluctuation amplitude and 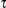, with 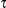 being an effective shear relaxation time. This emphasizes the dissipative (relaxational) aspect of 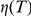, which is a distinctive feature of the present Markov network formulation. Such an interpretation is helpful to see how the model can be applied in practice, keeping in mind the essential characteristic of the model is the connectivity between the nodes, expressed by the inverse matrix **A** ^−1^ in Eq.(16). Matrix **A** is specified by a set of transition rates, 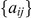, which are in turn defined by the activation energies 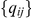 and the temperature. Thus our calculation of 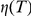 amounts to a determination of 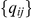 along with the nodal free energies {*F_i_*} which govern the probabilities {*P_i_*} in Eq.(16).

Note that the above derivation starts from Green-Kubo theory and assumes the system is in an equilibrium and ergodic condition among all *M* basins of the network. Calculations presented in this paper are performed under this assumption. However, the Network model can be extended to non-equilibrium conditions. One such implementation is discussed in the following section.

### Extension to Non-Equilibrium Systems

We show the Network model is not strictly limited to equilibrium liquids; with a simple modification it can be extended to compute the nonequilibrium viscosity of glass. The extension is based on treating the system as a broken ergodic system [10], [11] wherein the energy landscape is partitioned into sub-regions or “metabasins” satisfying the conditions of internal ergodicity (i.e., fast transitions within the metabasin) and confinement (i.e., slow transitions between metabasins).

The statistical mechanical treatment of broken ergodic systems comes in two basic flavors: discrete and continuous. The original discrete formulation by Palmer [10] considers a sudden breakdown of ergodicity where transitions between metabasins are strictly forbidden. This requirement is relaxed in the later treatment of Mauro *et al.* [12], [13], [14], who generalize the Palmer approach to account for a *continuous* breakdown of ergodicity at the glass transition. Since the laboratory glass transition is never a discontinuous process (i.e., an infinitely fast quench is never achievable in practice), the continuous formulation is more descriptive of realistic laboratory conditions. We will thus proceed in generalizing the Network model within the framework of continuously broken ergodicity (CBE).

Following the approach of Mauro *et al.* [12], [13], [14], [15], [16], the nonequilibrium dynamics of *P_i_*(*t*) can be computed for any thermal profile, *T*(*t*), by solving a system of master equations:
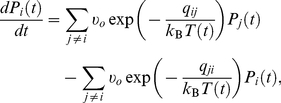
(18)where the initial condition is given from equilibrium statistical mechanics,
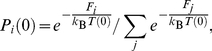
(19)and the transition rates are dependent on the energy barriers *q_ij_* and the instantaneous temperature. Taking advantage of the CBE formalism, the master equations can be solved on any arbitrary time scale through a dynamic partitioning of the landscape into metabasins satisfying the abovementioned criteria. The partitioning itself depends on three factors: (a) the topography of the landscape, (b) the instantaneous temperature, and (c) the observation time (inversely proportional to *dT/dt*). After partitioning, Eq. (18) can be rewritten in terms of a reduced set of master equations between metabasins (instead of between individual basins), allowing for solution of Eq. (18) on any arbitrary time scale. A complete discussion of this technique can be found in Refs. [12], [13], [14], [15], [16], including the calculation of *P_i_*(*t*) for a realistic glass-forming system (viz., selenium) using cooling rates from 10^−12^ to 10^12^ K/s.

With the above approach, Eq. (16) can be written in completely general form as:
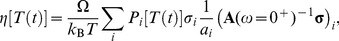
(20)where the equilibrium formulation is recovered in the ergodic limit. With this equation, one can study the effects of thermal history on the nonequilibrium viscosity of glass accounting for the continuous breakdown of ergodicity at the glass transition and the spontaneous relaxation to equilibrium. The subject of the nonequilibrium viscosity of glass is the subject of a thorough experimental and theoretical treatment in a separate paper by Mauro, Allan, and Potuzak [17].

### The Concept of Terrain and Network Model Calculation

In Eq.(16) the viscosity is resolved as a shear stress correlation, the product of two “inherent shear stresses 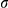
” modulated by a propagator **A**. The calculation of 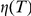 therefore amounts to a determination of 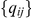 along with the nodal energies {*E_i_*} which govern the probabilities {*P_i_*} in Eq.(16).

**Figure 2:**
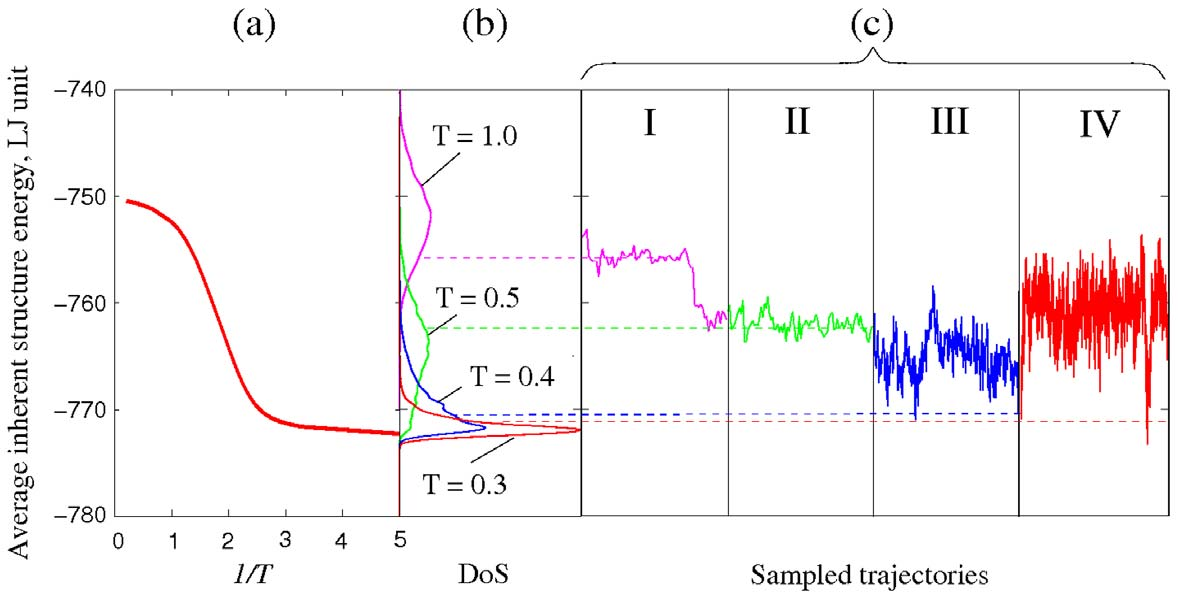
Data used in the Network model calculation. (a) Average inherent structure (IS) energy of BLJ liquid as a function of temperature, (b) Distributions of IS energies at four temperatures, 1.0, 0.5, 0.4, and 0.3, (c) Four TSP trajectories initialized at different energy minima (right panel).

While Eq.(16) is exact, in actual calculations one does not have access to the entire energy landscape, and therefore finite sampling of the landscape topography must anyhow be performed. While the potential energy surface (PES), *V*(**x** ^3*N*^), is temperature-independent, a key insight from previous molecular simulations is that a liquid experiences different “parts” of the same PES at different temperatures [18], [19]. This is like saying that while the Sahara and the Himalaya are both parts on the same planet, they have very different local “terrains”. Depending on the temperature, a liquid's phase-space trajectory travels in different typical “terrains”, and in evaluating Eq.(16) it is not necessary nor possible to feed the entire Earth's topography into it, but just a typical terrain of the “Sahara” or the “Himalaya” corresponding to that specific temperature. Such a typical “terrain” concept, a coarse descriptor of the actual PES being experienced, is intuitive to any traveler. Sastry, Debenedetti and Stillinger characterized the temperature-dependent terrains by the average valley bottom energy [18] (our Fig. 2a). Sciortino, Kob and Tartaglia characterized the degeneracy distribution of valley bottom energies by a temperature-dependent “inherent structure entropy”, from which they extracted the Kauzmann temperature *T*_K_ to be 0.3 [19].

## Results

### Viscosity Calculated by Network Model

As a practical approximation, we feed the transition state pathway (TSP) trajectories [5], [6] to Eq.(16), as a representative terrain for a given temperature. Recall how a system is prepared for TSP trajectory sampling. We start with a periodic simulation cell with *N* particles and an appropriate thermostat for MD simulation. After the system is equilibrated in the liquid state, it is supercooled to a temperature *T* below the melting point. Then MD simulation is continued at *T* during which a series of steepest descent relaxations is performed to obtain a distribution of energy minima (the inherent structure) and corresponding atomic configurations [20]. From this distribution an initial state for TSP trajectory sampling is selected (with a certain local minimum energy and associated atomic configuration). Each trajectory that is generated by the activation-relaxation sampling algorithm [5] therefore corresponds to a temperature *T* and an initial state (energy *E*_o_). Four such trajectories, generated at *T* = 0.5 and different initial states, are shown in Fig. 2 (see panel (c)) along with inherent structure calculations at the same *T*. All the results in the present work are obtained using the Kob-Andersen interatomic potential [21] for a binary Lennard-Jones (BLJ) model liquid adopted in Ref. [5]. Temperatures are expressed in reduced units.

In Fig. 2 we see in panel (a) the well-known temperature variation of the average inherent structure 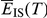 [18]. It is useful for interpretation purposes to regard 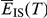 as the average well depth of the local energy minimum that the system on the average encounters at temperature *T*. In the liquid or barely supercooled liquid, 1/*T*<1.0, the wells are shallow. As the system is supercooled further, 1.0<1/*T*<3, the wells become deeper and reach a maximum depth when 1/*T*>3. The distribution of well depths at four temperatures (*T* = 1.0, 0.5, 0.4, and 0.3) are shown in Panel (b). They are broad at high temperatures, becoming narrow with greater supercooling, and narrows abruptly in the range 0.4<*T*<0.3, which is close to the inflection point in 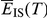. Correlated with the behavior in Panels (a) and (b) are the four TSP trajectories sampled at progressively lower energy initial states, labeled I, II, III, IV, in Panel (c). One sees the trajectories vary significantly with different *E*_o_. In Trajectory (I) which starts near the top of the inherent structure distribution the sampling gives small local minima and low activation energies. During the trajectory the system finds another minimum at significantly lower energy. This feature is not seen in trajectory (II), starting at a lower energy and apparently staying within the same energy range. In (I) and (II) the numbers of local energy minima sampled are 70 and 80 respectively. Trajectory (III), starting at still lower energy, is a larger sample size, 480 minima. It is seen to span a greater range of energy minima and activation energies which means sampling a larger region of the potential energy surface. Trajectory (IV) is the largest sample studied in this work at 3000 minima. Starting at a very low value of *E*_o_, its overall appearance shows significantly deeper minima and higher barriers. If we regard the trajectories as representative potential energy profiles, (IV) could serve as an example of a *rough* terrain in contrast to the small and relatively regular oscillations seen in (I) and (II).

Combining the inherent structure results with the sampled trajectories we anticipate that terrains (I) and (II) are suitable for the calculation of 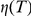 in the liquid and lightly supercooled states, whereas (IV) would be appropriate for the deeply supercooled states. On this basis we will use terrains (I) through (IV) in the following temperature ranges respectively, 1/*T*<1.25, 1.25<1/*T*<2, 2<1/*T*<2.5, 2.5<1/*T*.

In numerical calculations each Network node has the energy of a local minimum. The activation energy 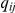 defines the transition rate 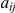, which in turn are used to construct the matrix **A**. For example, in using terrain (IV) we have a transition probability matrix of rank 3250. Each node has a given energy 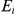, an occupation probability *P*_i_, and stress 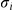. The viscosity is then calculated from Eq.(16).

**Figure 3:**
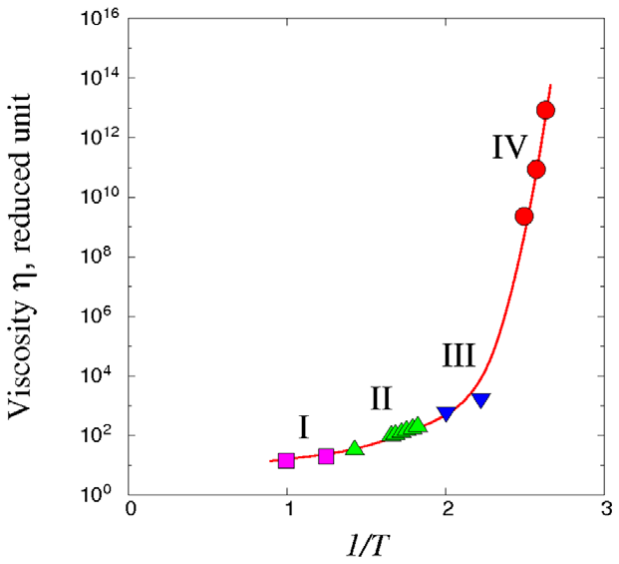
Viscosity computed using the Network model expression, **Eq. (16)**, with the four TSP trajectories shown in **Fig. 2** as input. Results for each trajectory are denoted by a different symbol, squares for trajectory I, triangle for II, inverted triangles for III, and circles for trajectory IV. Solid curve is a spline fit to all the calculated viscosities.

Fig. 3 shows the viscosities obtained using the four terrains in the corresponding temperature ranges specified above. The curve in Fig. 3 is a fit to the calculated values using a cubic spline. The fitting effectively serves as a coarse-grain average over the individual terrains. The results given by (I) and (II) are seen to connect smoothly with each other, with the first point from trajectory (III), and also with the results from (IV). As for the second point from (III) we believe the underestimate is an indication of insufficient sampling of the activation kinetics. The temperature variation of the viscosity over 12 orders of magnitude, seen in Fig. 3, is the essential prediction of our calculation. This is a composite result produced by combining the linear response (Green-Kubo) theory of transport as formulated in Eq.(16) for the binary Lennard-Jones interatomic potential model [21] with the four TSP trajectories, shown in Fig. 2, each of which specifies a propagator matrix **A** (see Eq.(16)) for a particular temperature range. We see the use of the four trajectories to calculate 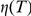 over the whole temperature range gives results that appear to be sound. In particular, high viscosity values correlate well with an energy landscape with large activation energies.

### Verification and Validation

**Figure 4:**
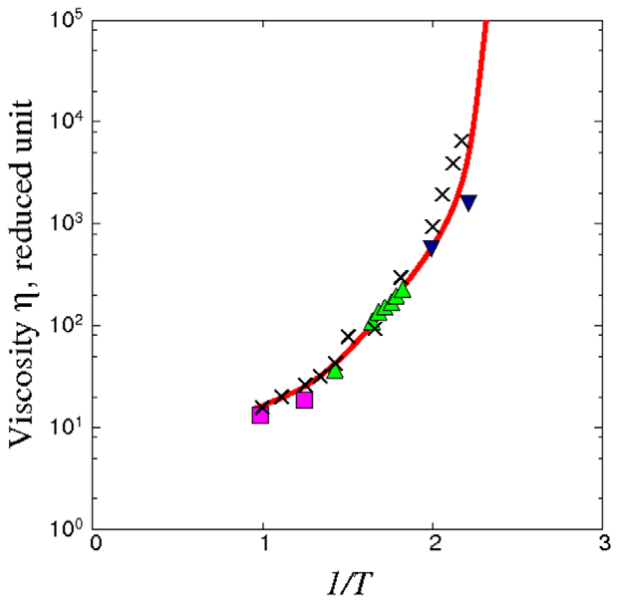
Comparison of Network model results (solid line) against Green-Kubo MD (crosses).

We first test the Network model by comparing the calculated viscosities with results obtained independently by direct MD simulation using the same interatomic potential. In Fig. 4 we see this test can be applied to trajectories (I), (II), and (III) because direct MD is able to reach viscosity values ∼10^4^. The good quantitative agreement provides verification of both Eq. (16) and the numerical implementation of the TSP trajectories in specifying the propagator matrix **A**.

**Figure 5:**
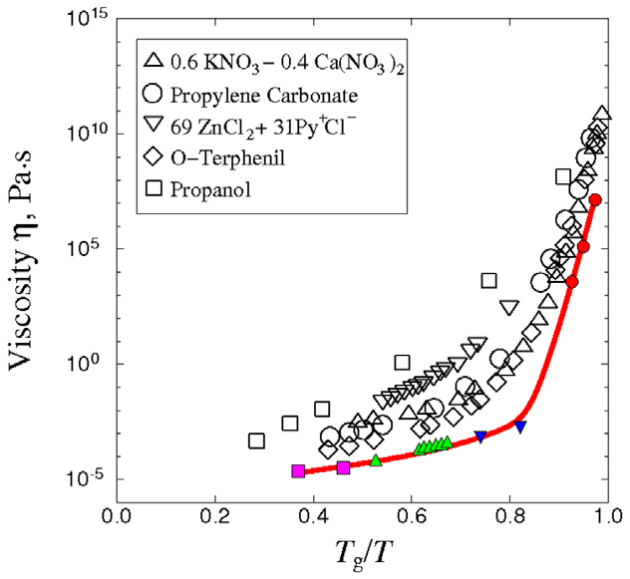
Experimental validation of the Network model. Solid line indicates the viscosity of BLJ liquid calculated by the Network model. Symbols are experimental data on fragile glass formers [3].

In the high-viscosity region, the only test available is by direct comparison with experimental data. Fig. 5 shows the comparison with measured viscosity in absolute unit for five liquids, where temperature is scaled by the glass transition temperature *T*_g_. For the Kob-Andersen potential we have determined *T*_g_ to be 0.37 (reduced unit) [5]. We see that overall the combination of Eq.(16) and use of TSP trajectories accounts quite well the observed temperature variation, from essentially Arrhenius at high temperatures (*T*_g_/*T*<0.7) through a temperature range where the viscosity variation is clearly super-Arrhenius (*fragile*). Comparing Figs. 2 and 5 we can interpret trajectory (III) as a representative energy landscape associated with the onset of fragile behavior. This kind of physical details, despite being fragmentary at present because of limited sampling, could lead to further insights into the dynamics of supercooled liquids. The agreement with experimental trend at low temperatures (*T* approaching *T*_g_) is noteworthy in that such viscosity magnitudes have not been reported in previous atomistic calculations.

**Figure 6:**
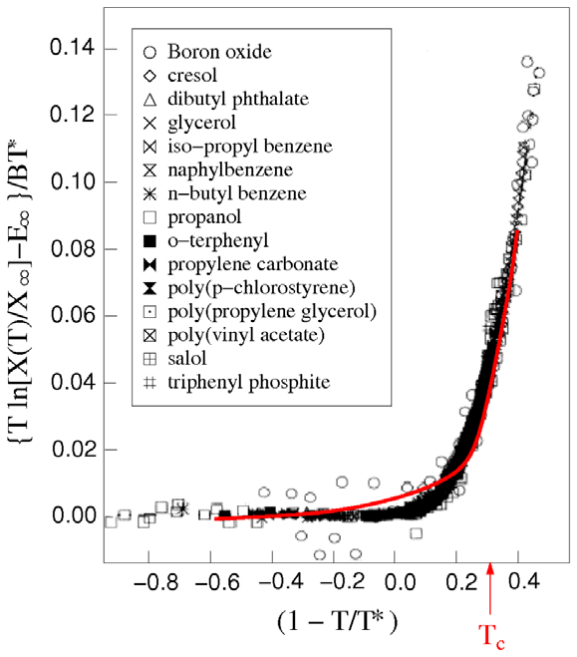
Comparison of an effective temperature-dependent activation barrier obtained from experimental data (symbols) [**4**] with similarly reduced results of the Network model (solid curve). The value of T* is 0.63 for the Network model.

Beyond direct comparison with individual measurements, additional experimental test can be made in terms of an effective temperature-dependent activation barrier [4]. In this case three parameters are involved in reducing the experimental data, scaling in temperature and viscosity, and normalization of barrier height. The experimental results for the activation barrier for a group of 15 liquids are shown in Fig. 6. They are seen to collapse onto a universal behavior.

Starting at high temperatures the barrier is a constant (normalized to zero) until the temperature reaches a characteristic value T*, where it begins to increase quite sharply. Notice that if one were to plot the quantity [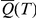
−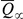] against (T*−*T*)/T*, the behavior would be very similar to plotting 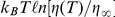 against *T*_g_/*T* (cf. Fig. 5). In Fig. 6 we also show the Network model calculations reduced in the same way. Previously a similar comparison was made with the results of a heuristic model (see Fig. 16 of Ref. [5]) rather than the present Network model results. Relative to the former significant improvement has been brought about by the latter; this is especially significant at low temperatures, *T*<T*. In this way of comparing calculation with experiments, fragile behavior begins at the onset of temperature-sensitive activation around the characteristic temperature T*. Since the value of T*, which is 0.63, is known from the scaling, we can compare T* with the so-called critical temperature *T*_c_ in mode coupling theory, where *T*_c_ = 0.435 (reduced unit) [21]. A slight discrepancy (overestimate) between Network model and experiments is seen in the lower barrier region (above *T*_c_).

## Discussion

Starting from the Green-Kubo theory [9] and solution of the master equation, we have developed an analytical expression for the viscosity of a material that is trapped in deep energy minima and makes infrequent hops in between. The system is assumed to be represented by a network of pair-wise coupled nodes (energy basins), each endowed with an inherent free energy and an inherent shear stress. The system evolves by hopping from one node (basin) to another according to a temperature-dependent transition probability specified by an activation free energy.

We then describe a quantitative study of the shear viscosity of a supercooled model liquid over a temperature from the onset of super-Arrhenius behavior down to *T*_g_. If we refer to the former as *T* ^*^, the value we find is approximately 0.63 (cf. Fig. 6). Because the Kob-Andersen model is well studied, we now have the values of several characteristic temperatures to serve as reference points in discussing the dynamics of supercooled liquids. The relevant temperature range includes the Kauzmann temperature *T*_K_ at 0.3 [19], *T*_g_ at 0.37, *T*_c_ at 0.435, and *T* ^*^ at 0.63. These values are seen to be consistent with each other considering the physical significance ascribed to each temperature. The numerical results and the comparisons with experiments discussed here suggest that the underlying TSP trajectories that would be representative for the temperature range *T*>*T*_c_, such as (I) and (II), are distinctly different for those in the range *T*<*T*_c_, such as (IV). This difference accounts for the different temperature variations observed experimentally. It also indicates that one could interpret a crossover temperature separating the region where the effects of barrier activation are not important from the region where such effects play an essential role. This observation, based on the results presented here, is fully compatible with the current understanding of mode coupling theory regarding the range of validity of its original formulation [22] and in an extended form which incorporates barrier hopping [23].

Our calculation is a master-equation approach that relies on potential energy landscape sampling to provide the appropriate transition rate matrix. Angelani and co-workers [24], [25] have studied the long time dynamics of a network system by analyzing the minima and saddles of small clusters, from 11 to 29 atoms. They showed the stress correlation displays a stretched exponential relaxation, and the Stokes-Einstein relation to breakdown at a temperature where the stretching exponent deviates from unity. We expect these characteristics to be found also in our Network model. On the other hand, Angelani et al. did not find the onset of fragile scaling behavior that we have seen in Figs. 5 and 6. Presumably one explanation is the absence of distributions of deep minima and large activation barriers in the energy landscape of small clusters.

We believe the most significant aspect of our study to be the calculation of viscosities in the range 10^8^ Pa·s and beyond. Since η is product of the shear modulus (∼10^10^ Pa) and a relaxation time, the implication is that atomistic simulation can approach time scales previously unimagined. The agreement with experiment that we find in Figs. 5 and 6 for the fragile liquids also extends to a “strong” liquid, silica, as shown in a less rigorous calculation than Eq. (16) which still makes use of the TSP trajectory [6]. This is encouraging evidence that the atomistic approach can be predictive.
